# Systematic identification of TPS genes in Gossypium and their characteristics in response to flooding stress

**DOI:** 10.3389/fpls.2023.1126884

**Published:** 2023-02-08

**Authors:** Aihua Cui, Yunqian Jin, Yongqi Li, Taili Nie, Liangqing Sun

**Affiliations:** ^1^ Scientific Research Office, Economic Crop Institute of Jiangxi Province, Jiujiang, Jiangxi, China; ^2^ College of Agronomy, Henan University of Science and Technology, Luoyang, China

**Keywords:** Cotton, terpene synthases, gene family, evolution, function

## Abstract

Terpene synthases (TPS) is a key enzyme in the synthesis of plant terpenoids. Studies on TPSs have not been reported in *Gossypium barbadense* and *Gossypium arboreum*. 260 TPSs were identified in Gossypium, including 71 in *Gossypium hirsutum*, 75 in *Gossypium. barbadense*, 60 in *Gossypium. arboreum*, and 54 in *Gossypium raimondii*. We systematically analyzed the TPS gene family of Gossypium from three aspects: gene structure, evolutionary process and gene function. (1) Gene structure: Based on the protein structure of two conserved domains (PF01397 and PF03936), the TPS gene family is divided into five clades: TPS -a, -b, -c, -e/f and -g. (2) Evolution: Whole genome duplication and segmental duplication are the main modes of TPS gene amplification. (3) Function: The abundance of cis-acting elements may reveal the functional diversity of TPSs in cotton. TPS gene has tissue specific expression in cotton. The hypomethylation of the exon of TPSs may help to enhance the adaptability of cotton to flooding stress. In conclusion, this study can broaden the understanding of structure-evolution-function of the TPS gene family, and provide reference for the mining and verification of new genes.

## Introduction

Terpenoids are the largest group metabolites in plant and respond positively to plant biotic and abiotic stresses (Cane, 2000; Yazaki et al., 2017; Huang et al., 2021).Terpenoids can be divided into broad categories according to their functions: primary metabolites and secondary metabolites. More than 55,000 members have been identified (Köksal et al., 2011). Previous work has shown that terpenoids are important in biodefense (Xiao et al., 2012; Irmisch et al., 2014; Alicandri et al., 2020; Huang et al., 2022), oxidation resistance (Xie et al., 2006), waterlogging resistance (Kuroha et al., 2018), and drought tolerance (Takahashi et al., 2018).

TPS is a critical enzyme for the production of plant terpenoids, mainly involved in the production of monoterpene, sesquiterpene and diterpene biosynthesis. Ancient TPSs originated in land plants that diverged from green algae (Jia et al., 2022). Studies have shown that TPS genes are involved in plant defense against pests and diseases and plant growth and development. *OsTPS19* enhanced the resistance to rice blast (Chen et al., 2018). TPS gene was involved in floral synthesis (Gao et al., 2018). *GhTPS10* was involved in the synthesis of gossypol (Huang et al., 2018). Based on the amino acid sequence and gene function of TPS, the TPS gene family was divided into seven clades: TPS-a, -b, -c, -d, -e/f, -g and -h (Chen et al., 2011). As more plant genomes are sequenced, TPS gene family members have been identified in a variety of plants. There were 29 TPSs in *Solanum lycopersicum* (Falara et al., 2011), 32 in *Setaria italica* (Karunanithi et al., 2020), 40 in *Arabidopsis thaliana* (Aubourg et al., 2002), 14 in *Selaginella tamariscina*, and 34 in *Oryza sativa* (Chen et al., 2011). The TPS gene family of *A. thaliana* was divided into five clades: TPS-A (22 TPSs), -b (6 TPSs), -c (1TPS), -e/f (2 TPSs), -g (1TPS) (Aubourg et al., 2002). At present, there were 41 and 46 TPSs in *Gossypium. raimondii* and *Gossypium. hirsutum*, respectively (Huang et al., 2018). Genome-wide identification and systematic analysis of the TPS gene family have not been reported in *Gossypium. barbadense* and *Gossypium. arboreum*.

Cotton is an important fiber crop. Flooding is a natural disaster frequently encountered during the seedling growth of cotton, which has a serious impact on cotton yield. At present, sequencing of *G. hirsutum* (TM-1, CRI-12), *G. barbadense* (Hai7124), *G. arboreum* and *G. raimondii* have been completed (Paterson et al., 2012; Du et al., 2018; Hu et al., 2019; Lu et al., 2019; Lu et al., 2022). It laid a good foundation for studying TPS gene in cotton. Studies suggested that *GhTPS12* may play a key role in cotton defense against herbivores (Huang et al., 2018). *GhTPS1*, *GhTPS2* and *GhTPS3* have been identified in *G. hirsutum* (Yang et al., 2013). The response of TPS to flooding in cotton has not been reported. This study was to broaden the understanding of the gene structure, phylogenetic evolution and gene function of TPSs.

## Materials and methods

### Download of database

Gene annotations and protein files for *G. arboreum* (Version 1.0, CRI), *G. raimondii* (Version 2.0), *G. hirsutum* (Version 2.1, ZJU), *G. barbadense* (Version 1.1, ZJU), and *A. thaliana* (TAIR10.) were obtained from online databases (https://cottonfgd.org/, http://www.arabidopsis.org/), respectively (Zhu et al., 2017).

### Identification of TPS

The Hidden Markov Model profiles for PF01397 and PF03936 can be acquired from the Pfam website. TPSs were retrieved from the cotton genome database using HMMER software. Redundant genes with e value greater than 1E-05 were deleted. TPSs with incomplete C and N terminus were deleted *via* the NCBI Batch CD-Search website (https://www.ncbi.nlm.nih.gov/). The transcription length and protein length of cotton TPS gene were further retrieved from CottonFGD (https://cottonfgd.org/) (Zhu et al., 2017).

### Phylogenetic analysis

The amino acid sequences of TPSs in five species are showed in the **Table S3**. The phylogenetic tree of TPS gene family was constructed using Neighbor-Joining (NJ) by MEGA 7.0. Bootstrap value:1000. (Larkin et al., 2007; Kumar et al., 2016).

### Location map of TPS

The location data of the GhTPS gene family on chromosomes were obtained from the genome annotation file. TBtools software was used to construct the map of TPS gene on chromosomes (Chen et al., 2020).

### TPS gene structure and protein motifs

Phylogenetic trees, motifs and structures were mapped by TBtools software using phylogenetic files (format: nwk), genome annotation files (format: gff3), and conserved motifs (format: MAST) in *G. hirsutum* (Bailey et al., 2009; Chen et al., 2020). The amino acid motif in the predicted GhTPS protein sequence was analyzed using online MEME website (https://meme-suite.org/meme/tools/meme). The sequence distribution site was set to 0 or 1, the ordinal number was set to 10, and all other parameters were set to default.

### TPS gene collinearity

MCScanX software was used to construct synteny relationships between duplicate gene pairs (Wang et al., 2012). We used TBtools software to display collinear maps (Chen et al., 2020).

### Selective pressure analysis

Duplicate gene pairs from four cotton species were identified by TBtools. The sequence identity after alignment should be higher than 80%. The non-synonymous (Ka) and synonymous (Ks) substitution ratio of duplicate genes were analyzed by TBtools software (Chen et al., 2020).

### Cis-acting elements and gene expression

PlantCARE website was used to predict the cis elements of GhTPS promoters (http://bioinformatics.psb.ugent.be/). Cis-acting elements were classified and analyzed. The RNA-Seq data was downloaded from the GRAND website (http://grand.cricaas.com.cn/home) (accession number: PRJNA490626). The relative expression patterns of GhTPS gene were analyzed at different time points (0, 1, 6 and 12 h) under PEG (200 g/liter), NaCl (0.4 M), cold (4 °C) and hot (37 °C) stress conditions (Yang et al., 2019). Methylation data download number: PRJNA856623.

### qRT-PCR

The GhTPS specific expression profiles in roots, stems and leaves and the response of TPS to flooding stress were analyzed by qRT-PCR. The experimental material was watering-resistant ZNL2067, which was grown in a light incubator at 25°C until the three-leaf stage. ZNL2067 was treated with flooding (3d) and reoxygenation (3d), and tissue samples were collected (three biological replicates per treatment). Total RNA was extracted according to the EASYspin Plus Plant RNA Kit instructions, then cDNA was synthesized according to the TransStart Top Green qPCR SuperMix Instructions manual. The primer sequence of GhTPS gene was shown in **Table S4**. The primer sequences of GhTPSs and Actin gene were shown in **Table S4**. Rapid fluorescence quantitative PCR was carried out on Bio-Rad 7500. We calculated the relative expression of GhTPS gene using 2^-ΔΔCt^ (Livak and Schmittgen, 2001).

## Results

### The acquisition of TPS gene members

**Figure 1:**
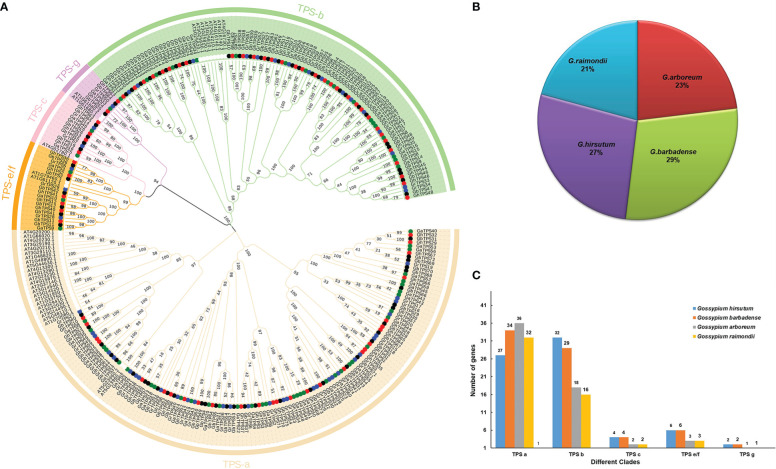
TPS gene family phylogenetic tree. **(A)** Phylogenetic relationships of 298 TPSs in five species. **(B)** The proportion of different cotton species in 260 TPS genes. **(C)** Distribution of gene number in five evolutionary clades. Different colors represent different clades.

298 TPSs were obtained from five species. 71, 75, 60, 54 and 38 TPSs were identified from *G. hirsutum*, G. *barbadense*, *G. arboreum*, *G. raimondii* and *A. thaliana*, respectively (**Figure 1**). Meanwhile, based on the physical location of TPSs on chromosomes, the four species TPSs were named as *GhTPS1*-*GhTPS71*, *GbTPS1*-*GbTPS75*, *GaTPS1*-*GaTPS60* and *GrTPS1*-*GrTPS54* respectively (**Table S1**). The TPS gene characteristics of cotton were further analyzed, including 16 indexes such as CDS length, exon number and protein length (**Table S2**).

In the model plant *G. hirsutum*, there were 71 TPS proteins ranging in length from 372 aa (*GhTPS45*) to 849 aa (*GhTPS12*). CDS Length (bp) ranged from 1119bp (*GhTPS37*) to 2550bp (*GhTPS12*). The isoelectric point ranged from 4.692 (*GhTPS37*) to 6.913 (*GhTPS61*). The number of exons ranged from 5 (*GhTPS45*) to 15 (*GhTPS26*). MW ranged from 43.341 (*GhTPS45*) kDa to 97.550 (*GhTPS53*) kDa.

### Phylogenetic analyses

To understand the evolutionary relationships of the TPS gene family among four cotton species, we constructed rootless phylogenetic trees of 298 TPS proteins (Saitou and Nei, 1987) (**Figure 1**). Based on the classification method of TPS gene proteins in *A. thaliana* (Jiang et al., 2019), 260 TPSs were divided into five evolutionary clades in Gossypium. The evolutionary clade TPS a contained the highest proportion of TPSs (129 cotton TPSs), and the distribution of the other four clades was as follows: TPS b (95 TPSs), TPS c (12 TPSs), TPS e/f (18 TPSs) and TPS g (6 TPSs) (**Figure 1C**; **Table S5**). TPSs were distributed in each clade. The ratio of diploid cotton to allotetraploid cotton was less than 1:2 (**Figure 1B**). This suggests that the loss of the TPS gene occurred during the formation of allotetraploid.

### Chromosomal location of TPS

**Figure 2:**
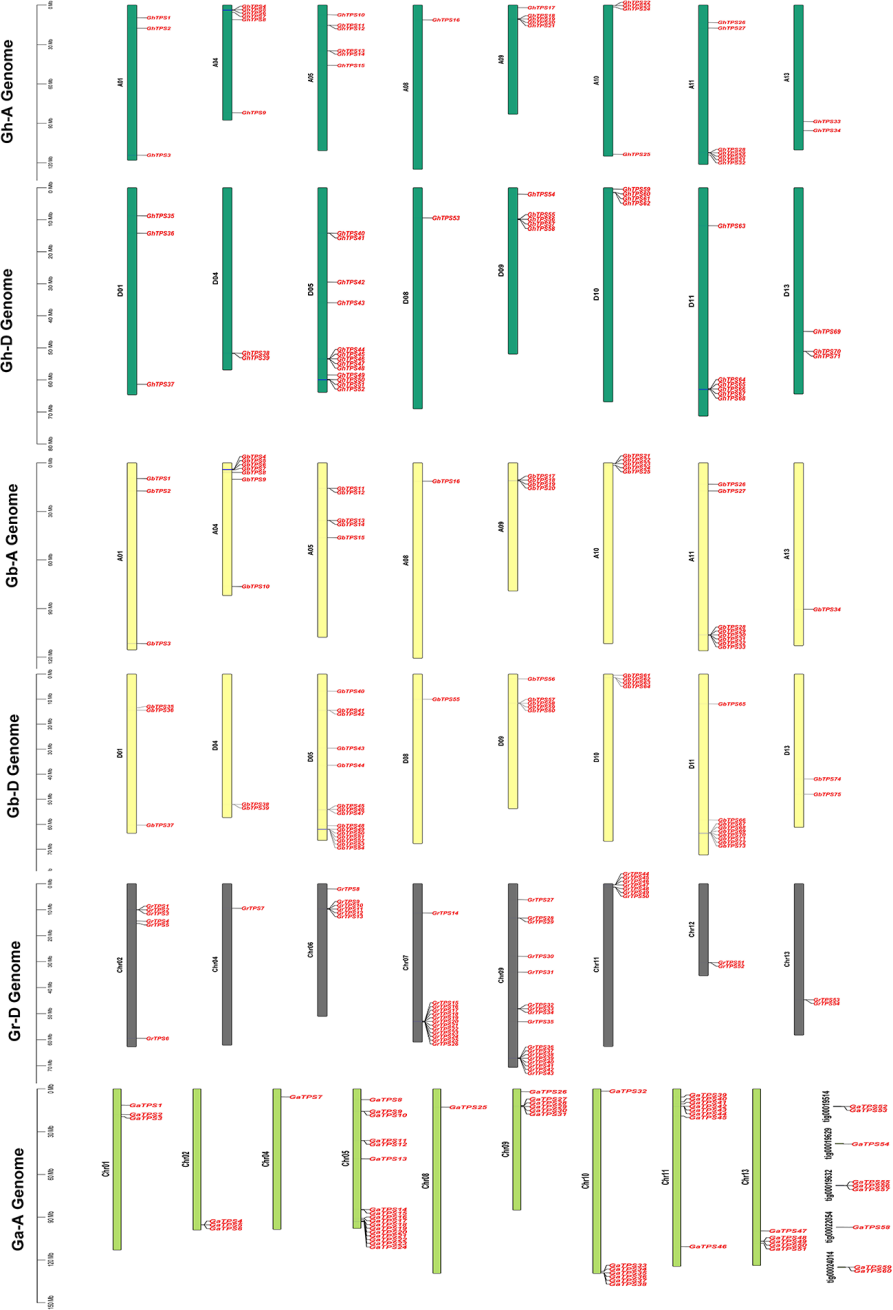
Chromosomal mapping of TPS in Gossypium. The vertical bars indicate the physical location of genes and the length of chromosomes. The gene names are on the right side of chromosomes. The four colors represent the four cotton species.

To study the physical location of TPSs on chromosomes, we constructed chromosome maps of 260 TPSs (**Figure 2**). 251 genes were assigned to specific chromosomes (**Figure 2**; **Table S6**). Among the 71 GhTPSs in *G. hirsutum*, 34 and 37 TPSs were located in the At and Dt subgenome, respectively (**Figure 2**). For At subgenome: GHAt-11 had the most members (7 GhTPSs). For Dt subgenome: Dt-05 has 13 GhTPSs (**Figure 2**; **Table S6**). 75 TPSs were mapped to specific chromosomes in *G. barbadense* **(** **Figure 2**). The At and Dt subgenome contained 34 and 41 GbTPSs, respectively. For At:subgenome, At-11 had the most TPS members (8 GbTPSs). For Dt subgenome: Dt-05 had the highest number of TPS members (15 GbTPSs). *G. hirsutum* and *G. barbadense* belong to allotetraploid cotton, while *G. arboreum* and *G. raimondii* belong to diploid cotton. Interestingly, no TPS gene was found in chromosomes At/Dt-02, 03, 06, 07, and 12 in the allotetraploid cotton (**Figure 2**; **Table S6**). 51 GaTPSs were annotated onto 13 chromosomes, and 9 GaTPSs were not annotated on the chromosome in *G. arboreum* (**Figure 2**). There were 17, 8 and 7 GaTPSs on chromosomes of Chr05 (A05), Chr11 (A11) and Chr09 (A09). No GaTPSs were found on Chr03 (A03), Chr06 (A06), Chr07 (A07), and Chr12 (A12) chromosomes (**Figure 2**; **Table S6**). For *G. raimondii*, all 54 GrTPSs were annotated on chromosomes (**Figure 2**). There were more GrTPSs on chromosomes of Chr09 (D09) and Chr07 (D07), 17 and 13, respectively (**Figure 2**; **Table S6**). TPSs were unevenly distributed on chromosomes of Gossypium.

### Structure and protein motifs analysis of GhTPS

**Figure 3:**
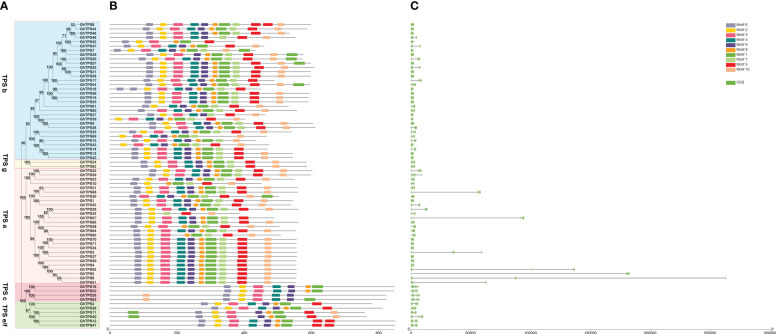
Phylogenetic tree - motif - structure of GhTPSs. **(A)** Phylogenetic tree. **(B)** Protein motifs. **(C)** Gene structures.

In order to characterize the protein motif and gene structure during the evolution of GhTPSs in *G. hirsutum*, we constructed a phylogenetic tree, conserved motif, and gene structure relationship map of TPSs (**Figure 3**). For protein motifs, GhTPS proteins had conserved motifs ranging from 5 to 10. The evolutionary clade TPS a had Motif 5, 6, 7. The evolutionary clade TPS c had Motif 2, 3, 4, 6, 9. The evolutionary clade TPS e/f had Motif 1, 2, 3, 5, 6, 8, 9. The evolutionary clade TPS g had 10 conserved motifs. The variation of the conserved motif of TPS b in the evolutionary clades suggested that it may have a wider range of biological functions. Similar protein motifs were found in the same evolutionary clade.

In the GhTPS gene family, the longest gene (*GhTPS6*) was approximately 263107 bp in length. *GhTPS43* was the shortest gene, 1839 bp. The number of exons in the GhTPS gene family was 6 ~ 15. (**Figure 3**). Among 71 GhTPSs, 44 genes had 7 exons. GhTPSs from the same evolutionary branch had similar genetic structure. It can be seen that the GhTPS gene family formed conserved gene structure and conserved motifs during evolution.

### Analysis of collinearity of the TPS gene family

**Figure 4:**
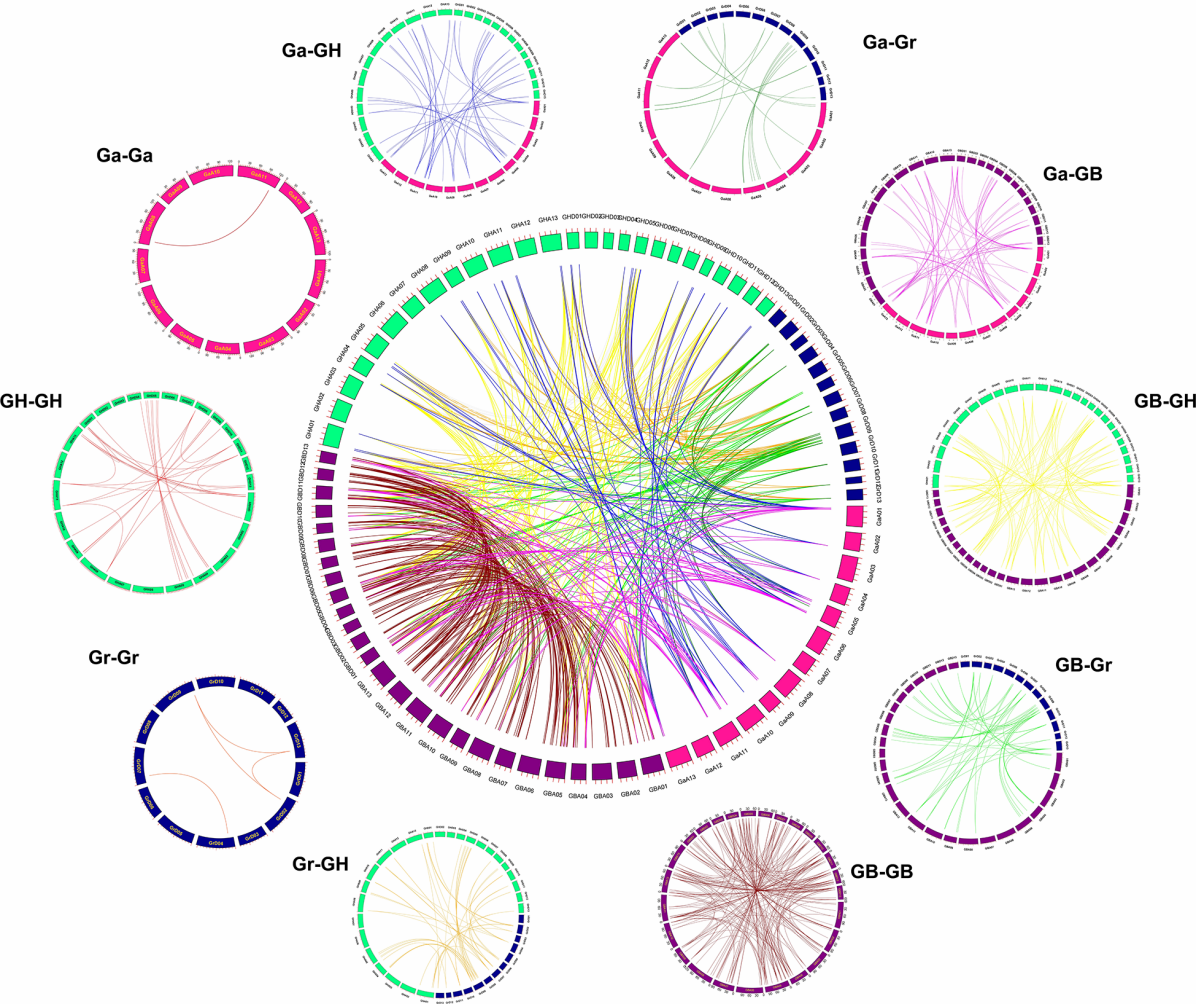
Syntenic relationship of 699 duplicated genes pairs in cotton. GHA, GHD, GBA, GBD, GaA and GrD represent At/Dt sub-genome of *G. hirsutum*, At/Dt sub-genome of *G. barbadense*, A genome of *G. arboreum* and D subgenome of *G. raimondii*, respectively.

**Figure 5:**
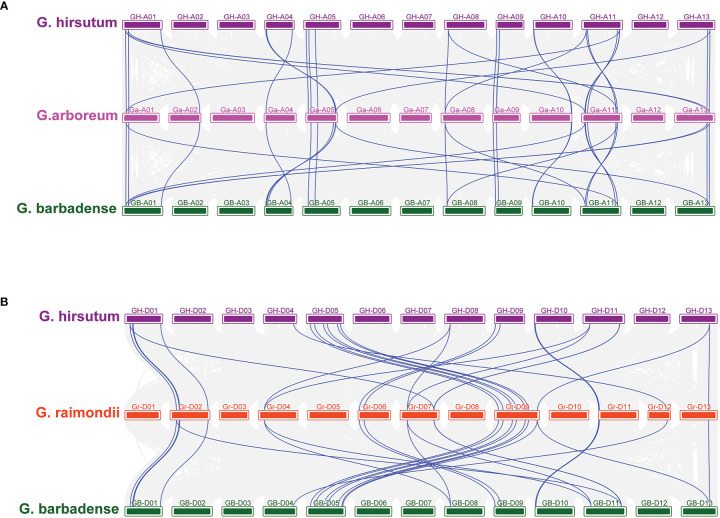
Collinearity between allotetraploid and its ancestral species **(A)** Collinearity of GhTPSs and GbTPSs compared with *G arboreum*. **(B)** Collinearity of GhGUT and GbTPSs compared with *G raimondii.* The blue line represents the TPS gene pair.

In order to explore gene amplification of the GhTPS gene family, a synteny/collinear relationship map of duplication gene pairs were constructed between the diploid ancestor A & D genome and the allotetraploid AD genome (**Figures 4**, **5**). There were 699 duplication gene pairs obtained in Gossypium including 239 segmental duplications and 51 tandem duplications. The whole genome duplication of the remaining 409 orthologous genes was performed (**Figure 4**). Taking *G. hirsutum* and *G. barbadense* as examples, 389 orthologous/paralogous gene pairs were obtained. There were 234 gene pairs that underwent segmental duplication. 35 and 120 gene pairs that were subjected to tandem duplication, and whole-genome duplication, respectively. It can be seen that genome multiploidy and segmental duplication are the primary modes of the TPS gene family amplification.

In order to understand the genetic amplification of A or D subgenome during evolution, we constructed collinear relationships between the GBAt-GaA-GHAt and GBDt-GrD-GHDt genomes. Chromosomes A01, A05, A11 and A13 had the most common linear genes from the A genome to the AD genome. However, D02, D07 and D09 of the D genome had a higher number of genes in common with the AtDt genome. The A genome had 58 and 76 duplicate gene pairs related to heterotetraploid AD genome, respectively. The D genome had 61 and 71 pairs of duplicated gene pairs associated with the heterotetraploid AD genome, respectively (**Figures 5**, **S1**).

### Selective pressure analysis

**Figure 6:**
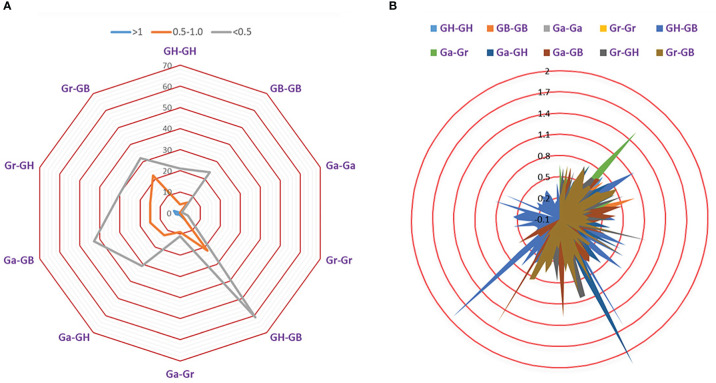
Selection pressure of TPS gene family. **(A)** The number of duplicate genes with different Ka/Ks values. **(B)** Ka/Ks divergence values of different genomes.

To explore the effects of selection pressure on the evolution of TPS gene family, Ks and Ka values of orthologous/paralogous pairs of four cotton species were calculated (**Figure 6**; **Table S7**).

There were 364 (96.81%) gene pairs with Ka/Ks < 1, 258 gene pairs with Ka/Ks < 0.5, and 106 gene pairs with Ka/Ks values ranging from 0.5 ~ 0.99. This revealed that the TPS gene family is highly conserved and has been subjected to strong purifying selection during evolution. The Ka/Ks ratio of 12(3.19%) orthologous/paralogous pairs was greater than 1, suggesting that TPS family may have experienced positive selection pressure during the process of chromosome doubling. The Ka/Ks for Ga-Ga and Gr-Gr were both less than 1, which indicated that TPS gene of diploid cotton was strongly conserved. As a result, we speculated that the cotton TPS gene family is an ancient family that has experienced strong purification selection pressure during the long evolutionary process (**Figures 6A, B**; **Table S7**).

### Gene enrichment analysis

**Figure 7:**
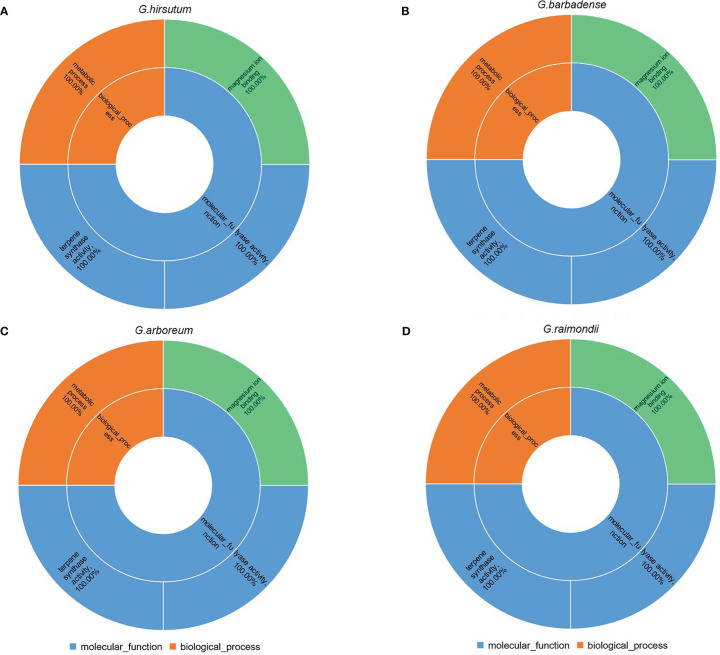
GO analysis of 260 TPSs. **(A–D)** represents GO enrichment of *G. hirsutum*, *G. barbadense*, *G.arboreum*, *G. raimondii*, respectively.

We predicted the function of 260 TPSs by gene ontology (GO) analysis in cotton. GO analysis indicated that TPSs were mainly participated in molecular functions and biological processes in cotton (**Figure 7**; **Table S8**). The 260 TPSs were involved in molecular functions including: terpene synthase activity, lyase activity, magnesium ion binding. For biological processes, TPSs were fully annotated to metabolic processes (GO:0008152). Therefore, TPSs play essential role in metabolism in cotton.

### Analysis of GhTPS cis-acting element

**Figure 8:**
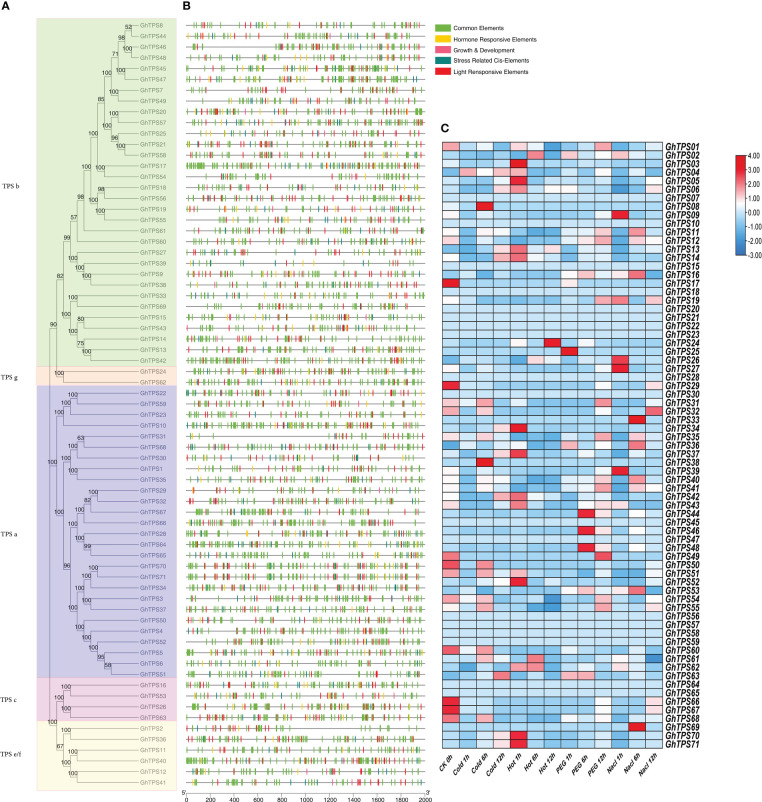
Expressed genes analysis and cis-acting elements of the GhTPS gene family. **(A)** Phylogenetic tree of GhTPSs. **(B)** Cis-elements of GhTPSs. **(C)** Expressed analysis of GhTPSs at different time points (0, 1, 6, 12h) under cold, hot, salt, PEG (FPKM).

Prediction and analysis of promoter region cis-acting elements can infer the function of downstream genes. The types and number of cis-acting elements from the same evolutionary clade were different (**Figure 8A**). The number and type of cis-acting elements in the same evolutionary clade were different. DNA sequences upstream of transcription initiation sites (TTS) in 71 TPSs were analyzed. There were 23 kinds of cis-acting elements associated to light reaction. Box4, G-Box and GT1-motif accounted for 94%, 83% and 72% of the total GhTPSs, respectively (**Figure 8B**; **Table S9**). Eight cis-acting elements were identified in response to biological/abiotic stress, with ARE being the most abundant (**Figure 8B**; **Table S9**). Ten cis-acting elements, including ABRE, CGTCA-motif, and TGACG-motif, are associated with plant prohormone responses. They accounted for 83%, 63%, 63% and 51% of the total GhTPSs, respectively (**Figure 8B**; **Table S9**). Similarly, 10 cis-acting elements were related to growth and development, AT-rich elements were the most abundant, accounting for 30% of the total GhTPSs. It is speculated that the GhTPS gene family has a critical role in plant growth process and in in biotic and abiotic stress responses.

Based on RNA-Seq data of *G. hirsutum* (TM-1, accession number: PRJNA490626), we examined the specific expression pattern of GhTPS in response to NaCl (0.4 M), PEG (200 g/liter), heat (37°C) and cold (4°C) stresses (**Figure 8C**; **Table S10**). Under different abiotic stress, *GhTPS54*, *GhTPS55*, *GhTPS70*, *GhTPS71*, *GhTPS12*, *GhTPS13*, *GhTPS40*, *GhTPS41*, *GhTPS42* and other genes showed different expressions. For example, *GhTPS55*, *GhTPS70* and *GhTPS71* were differentially expressed under cold stress. *GhTPS 42*, *GhTPS55* and *GhTPS70* were differentially expressed during heat treatment. Interestingly, *GhTPS55* gene was highly expressed in all of the above stress situations.

### Tissue-specific expression profile of GhTPS

**Figure 9:**
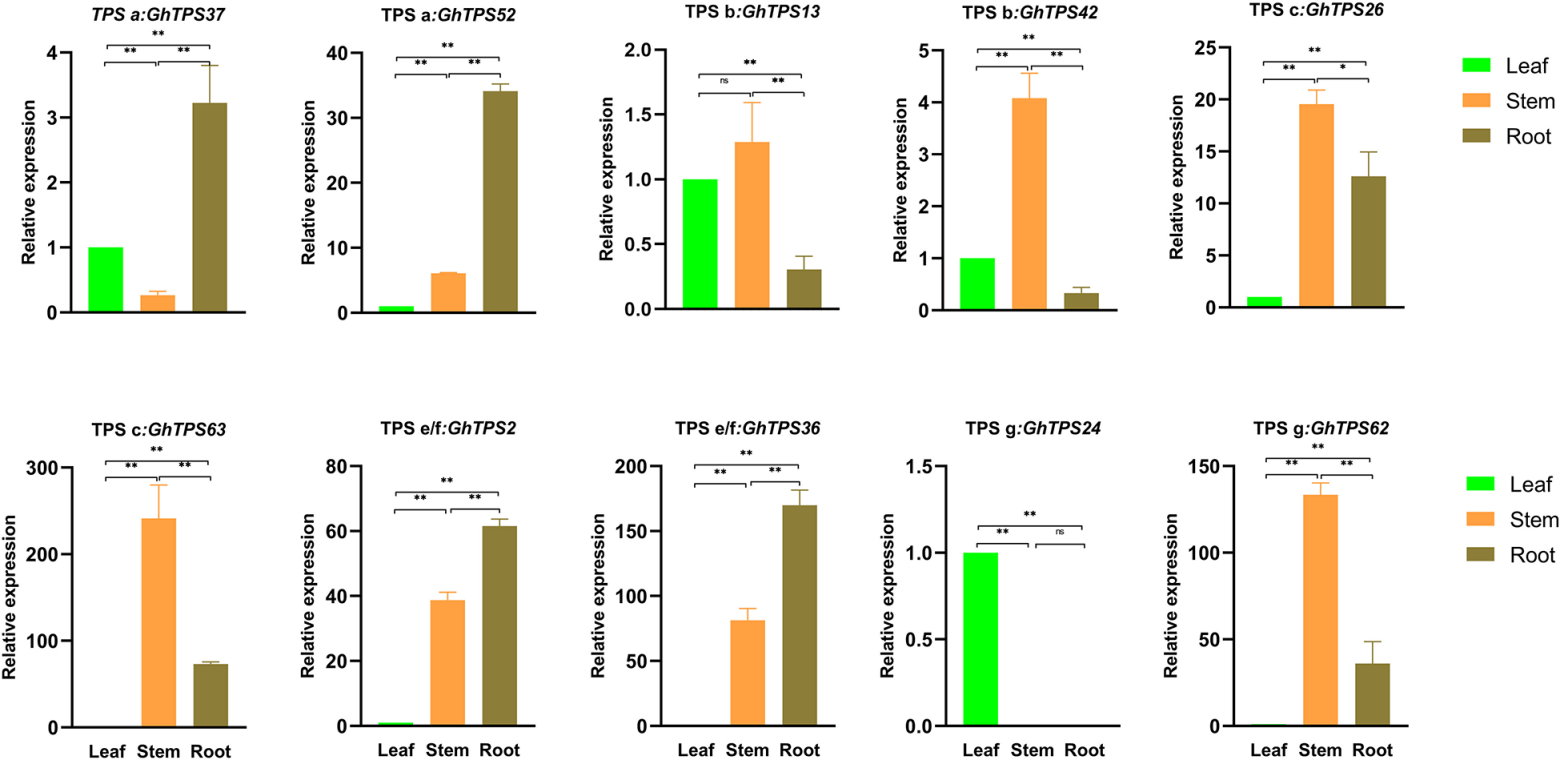
Tissue-specific expression of 10 GhTPSs. Error bars are the standard deviation (SD) of biological replicates. ns, p>0.05, *0.05>p>0.01, **p<0.01.

To further understand the tissue-specific expression profile of the GhTPSs and their reaction to flooding stress, we analyzed 10 GhTPSs from five clades. The expression profile of 10 GhTPSs were different in roots, stems and leaves (**Figure 9**). For example, *GhTPS24* was only highly expressed in leaves. *GhTPS42*, *GhTPS62* and *GhTPS63* were expressed at high levels in the stem. *GhTPS37* and *GhTPS62* were strongly expressed in the root (**Figure 9**).

### Analysis of TPSs response to flooding stress

**Figure 10:**
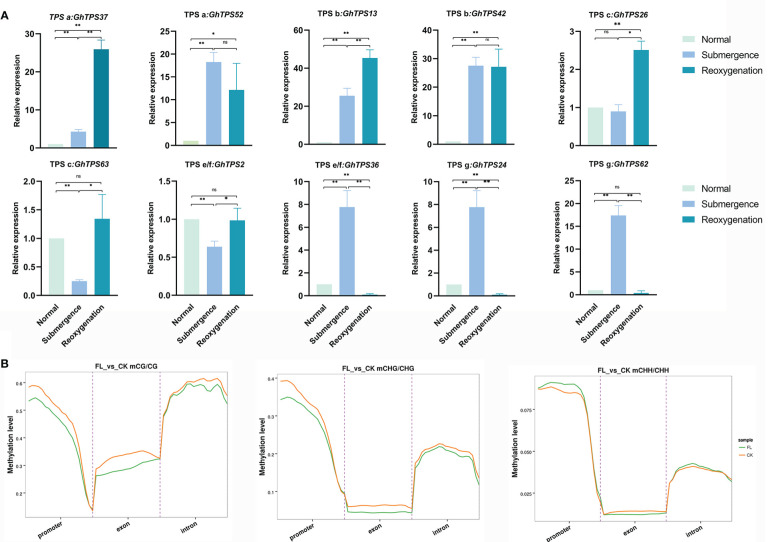
Levels of methylation in the CG/CHG/CHH sequences and expression levels of GhTPSs. **(A)** The expression levels of 10 GhTPSs under submergence and reoxygenation stresses. Error bars represent SD in biological replicates. **(B)** Distribution of methylation levels in functional region. Different colors represent groups. ns, p>0.05, *0.05>p>0.01, **p<0.01.

The expression levels of GhTPSs in response to flooding stress were different (**Figure 10A**). For example, *GhTPS36*, *GhTPS24* and *GhTPS62* were strongly expressed during submergence. *GhTPS36*, *GhTPS24* and *GHTPS62* were expressed at high levels under submergence and reoxygenation stress. *GhTPS37* was highly expressed under reoxygenation stress. There were also differences in tissue specific expression and response of GhTPSs in the same clade to flooding stress.

Under flooding stress, the methylation levels of CG and CHG sequences decreased in the promoter and introns region, while the methylation levels of CHH sequences increased. The methylation levels of CG, CHG, and CHH sequences in the exon domain were all reduced (**Figure 10B**). The hypomethylation of the exon of TPSs may help to enhance the adaptability of cotton to flooding stress.

### GhTPS protein interaction

**Figure 11:**
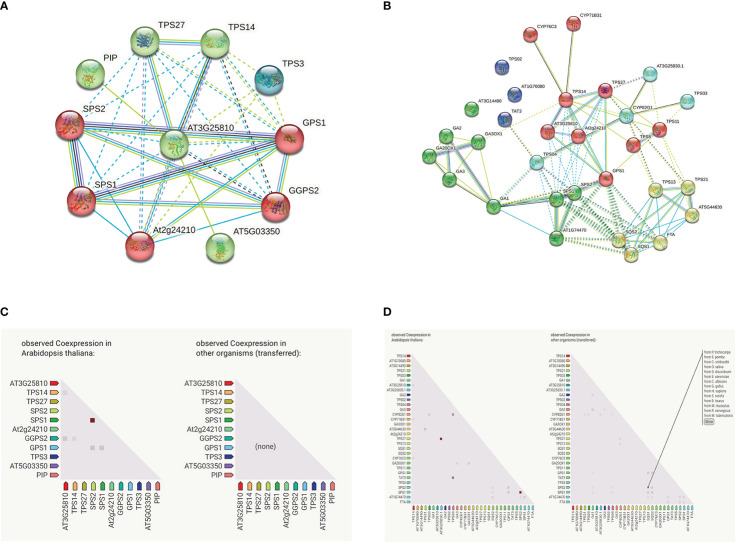
GhTPS proteins interaction network. **(A)** *GhTPS*42 protein interaction network. **(B)** Interaction network of GhTPS proteins. **(C)** *GhTPS42* gene co-expressed. **(D)** Gene co-expression of the GhTPS gene family. Note: The homologous gene of *GhTPS42* in *A thaliana* is AT3G25810.

Based on the homologous gene profile of *A. thaliana*, we predict GhTPS protein function through an interactive network using the online STRING website (https://string-db.org/) (**Figure 11**). In the bological process, 30 GO-terms were significantly enriched, such as plastoquinone biosynthetic process (GO:0010236), monoterpene biosynthetic process (GO:0043693), terpene biosynthetic process (GO:0046246), etc. In terms of molecular function, 20 GO-terms were significantly enriched, such as trans-octaprenyltranstransferase activity (GO: 0050347), sesquiterpene synthase activity (GO:0010334), (E)-beta-ocimene synthase activity (GO: 0034768), etc. In terms of molecular composition, chloroplast (GO:0009507), cytoplasm (GO:0005737) and cellular anatomical entity (GO:0110165) were significantly enriched in GO-terms (**Table S11**). In the KEGG pathway, it mainly involved monoterpenoid biosynthesis (ath00902), diterpenoid biosynthesis (ath00904), sesquiterpenoid and triterpenoid steroid biosynthesis (ath00909), terpenoid backbone biosynthesis (ath00900), steroid biosynthesis (ath00100), biosynthesis of secondary metabolites (ath01110). At the same time, we analyzed the protein interaction network of *GhTPS42* gene that was significantly up-regulated under flooding treatment (**Figure 11**).

## Discussion

Different plants contain different numbers of TPSs. 260 TPSs were identified in Gossypium (71 in *G. hirsutum*, 75 in *G. barbadense*, 60 in *G. arboreum* and 54 in *G. raimondii*. More TPSs were found in Gossypium than in *Solanum lycopersicum* (Falara et al., 2011), *Setaria italica* (Karunanithi et al., 2020), *A. thaliana* (Aubourg et al., 2002), *Selaginella tamariscina*, and 40 in rice (Chen et al., 2011). Previous studies identified 41 and 46 TPSs from *G. raimondii* and *G. hirsutum*, respectively (Huang et al., 2018), while we identified 54 and 71 TPSs, which were related to the different reference genomes we used. In recent years, with the increasing improvements in sequencing technology, the annotation of reference genome is more perfect, and the identification of gene family members is more accurate.

### Structural analysis of TPS gene family

The physical location of a gene on a chromosome affects its biological function. We found that TPSs were not uniformly distributed in chromosomes and usually existed in the form of gene clusters. Genes in a gene cluster usually belong to the same evolutionary clade. A series of 51 tandem repeat gene pairs were discovered on this chromosome. The number and sequence of exons in a gene are associated with its biological function (Malik et al., 2020). For *G. hirsutum*, we found that the length of GhTPS gene varied widely, from 1839 bp to 263107 bp. The number of exons varies from 6 to 15. These indicated that the gene structure of GhTPS was diversified. At the same time, GhTPSs from the same evolutionary clade had similar gene structures and protein motifs (**Figure 3**).

### Evolutionary analysis of the TPS gene family

To understand the changes in the TPS gene family over the long term, we analyzed the phylogenetic trees and selection pressures of TPS gene families in four cotton species. 260 TPSs were assigned to five clades, namely TPS - a, -b, -c, -e/f and -g. Previous studies have shown that TPS a is the largest clade in most dicotyledons and monocotyledons (Jiang et al., 2019). We also found that the evolutionary clade TPS a contained the largest number of TPSs (**Figure 1**; **Table S5**). TPS d is an endemic clade of gymnosperm (Bohlmann et al., 1998). The TPS h clade is identified only in the *Selaginella tamariscina* (Chen et al., 2011). TPS d and TPS h were not found in four cotton species.

Substantial gene amplification contributes to the formation of new species and adaptation to adversity (Hittinger and Carroll, 2007; Conant and Wolfe, 2008). Whole genome duplication, segmental duplication and tandem duplication are essential pathways for gene amplification. Cotton is one of the model crops studied for polyploidization (Li et al., 2015). A total of 699 duplicate gene pairs were obtained in Gossypium, including 409 whole genome duplication genes, 239 segmental duplication genes and 51 tandem duplication genes (**Figure 4**). Therefore, the three gene amplification modes played an important role in the amplification of the TPS family in Gossypium.

Previous studies have shown that heterotetraploid cotton is produced by interspecific hybridization of A and D genomes (Wendel and Cronn, 2003; Paterson et al., 2012; Li et al., 2015). The number of TPSs from allotetraploid cotton was less than twice that of diploid cotton, which might be due to gene deletions during the evolutionary process of forming allotetraploid cotton. Gene loss is also present in the evolution of the GRX, AHL and UGT gene families (Malik et al., 2020; Zhao et al., 2020; Sun et al., 2022).

When Ka/Ks>1, the TPS family was subjected to positive selection in the long-term evolution process. When Ka/Ks=1, the TPS family was subject to neutral evolution. When Ka/Ks<1, the TPS family was subjected to purify selection in the long-term evolution process. Our results showed that 96.81% of TPS gene pairs (364) had Ka/Ks values less than 1, which indicates that the TPS gene family experienced highly purified selection pressure over the long evolutionary period (**Figures 6A, B**; **Table S7**). Meanwhile, 12 (3.19%) gene pairs had Ka/Ks values greater than 1, which demonstrates that TPSs underwent positive selection pressure after gene replication.

### Functional analysis of the TPS gene family

The results showed that 10 cis-acting elements were involved in cotton growth and development, and AT-rich element was the most involved. 10 cis-acting elements were related to regulate plant hormone; AT-rich elements were the most. There were also differences in the cis-acting elements of genes within the same evolutionary clade. At the same time, we found that GhTPS gene has tissue specific expression characteristics. For example, *GhTPS24* was expressed at high levels in leaves, while *GhTPS42*, *GhTPS62* and *GhTPS63* were expressed at high levels in stems (**Figure 9**). In general, TPSs are essential in the cotton growth and development.

Eight cis-acting elements responded to biological/abiotic stress of cotton, among which ARE and LTR were more (**Table S9**). Under different stress treatments, not all genes had biological functions. For example, *GhTPS55* gene was highly expressed in different stress situations, while many TPSs were not involved in stress response. Previous studies have also verified this conclusion. In some angiosperms and gymnosperms, not all TPSs are functional (Chen et al., 2011). Through comprehensive analysis of expression profile, cis-regulatory elements and protein interaction, TPS gene may play an important role in waterlogging stress.

## Conclusion

**Figure 12:**
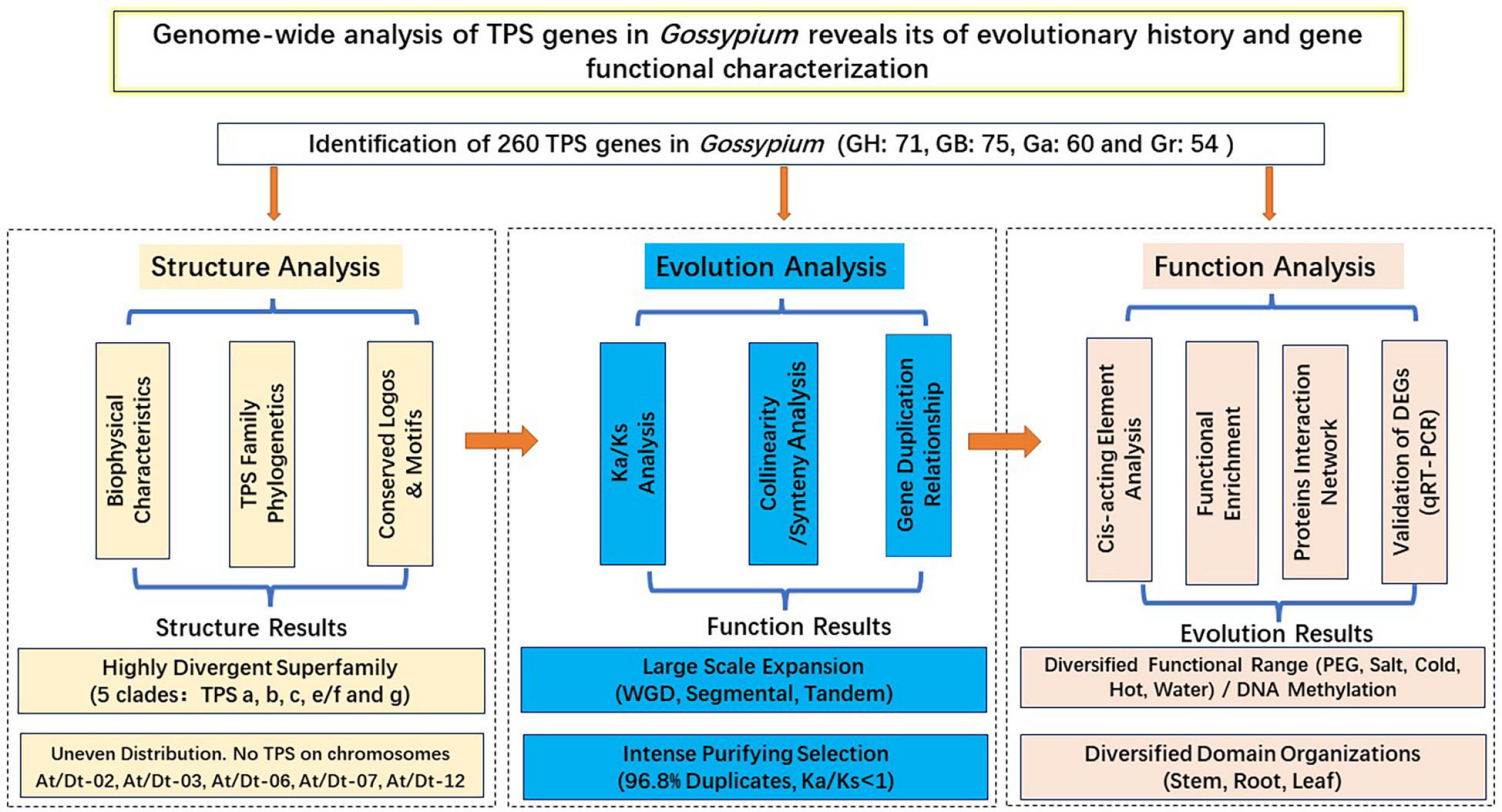
Genome-wide and systematic analysis of the TPS gene family in Gossypium.

TPS is a critical enzyme for the production of plant terpenoids, mainly involved in the production of monoterpene, sesquiterpene and diterpene biosynthesis. A total of 260 TPSs were identified, including 71 in *G. hirsutum*, 75 in *G. barbadense*, 54 in *G. raimondii* and 60 in *G. arboreum*. We systematically analyzed the TPS gene family of Gossypium from three aspects: gene structure, evolutionary process and gene function (**Figure 12**). (1) Gene structure analysis: Based on the protein structure of two conserved domains (PF01397 and PF03936), the TPS gene family was divided into five clades: TPS -a, -b, -c, -e/f and -g. (2) Evolution analysis: Genome multiploidy and segmental duplication are the main ways of TPS gene amplification. The TPS gene family underwent strong purification selection pressure during the long evolutionary process. (3) Function analysis: The abundance of cis-acting elements may reveal the functional diversity of TPSs in cotton. Cotton TPS gene is tissue-specific and plays an important role in stress. TPS gene has tissue specific expression in cotton and plays a key role in stress. In conclusion, this study can broaden the understanding of structure-evolution-function of the TPS gene family, and provide reference for the mining and verification of new genes.

## Data availability statement

The datasets presented in this study can be found in online repositories. The names of the repository/repositories and accession number(s) can be found in the article/**Supplementary Material**.

## Author contributions

Conceived and designed the experiments: LS and TN; methodology: AC and YJ; experiment: AC, YJ, and LS; analysis of data: LS and YL; writing-original draft preparation: AC and YJ; writing-review and editing: LS; supervision: TN and LS. All authors contributed to the article and approved the submitted version.
