# Supplementary figures and images for: Systematic identification of TPS genes in Gossypium and their characteristics in response to flooding stress
Source: Front Plant Sci. 2023 Feb 8;14:1126884. doi: 10.3389/fpls.2023.1126884 (PMC9945120; doi:10.3389/fpls.2023.1126884)

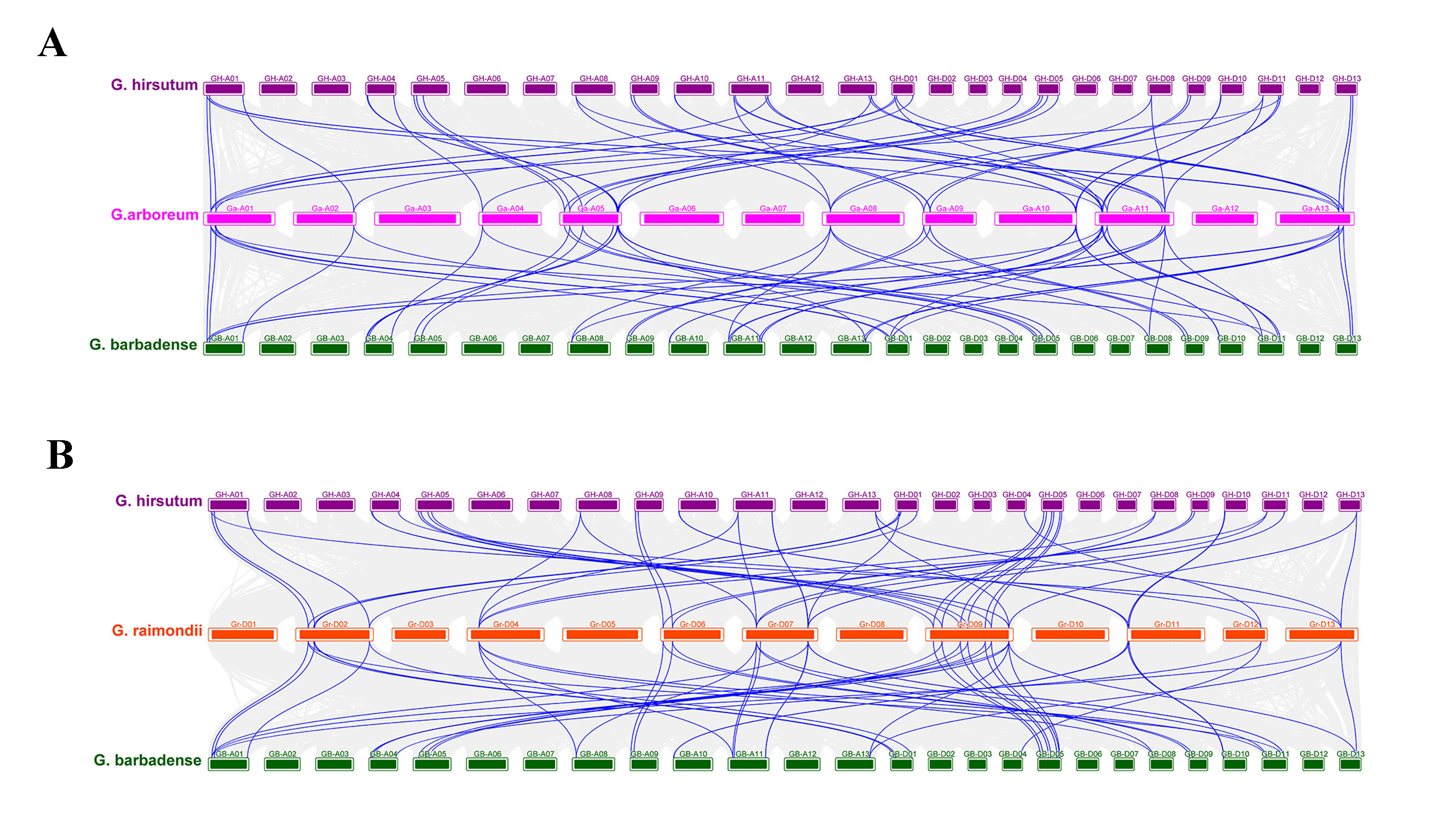

Supplement: Supplementary file 1 [file Image_1.jpeg]
